# Evaluation of a Multi-Gene Methylation Blood-Test for the Detection of Colorectal Cancer

**DOI:** 10.3390/medsci11030060

**Published:** 2023-09-15

**Authors:** Joel Petit, Georgia Carroll, Henry Williams, Peter Pockney, Rodney J. Scott

**Affiliations:** 1 Division of Surgery, John Hunter Hospital, Newcastle, NSW 2305, Australia; 2 School of Biomedical Sciences and Pharmacy, University of Newcastle, Newcastle, NSW 2305, Australia; 3 Hunter Medical Research Institute, University of Newcastle, Newcastle, NSW 2305, Australia; 4 School of Medicine and Public Health, University of Newcastle, Newcastle, NSW 2305, Australia; 5 Pathology North, Newcastle, NSW 2305, Australia

**Keywords:** circulating tumour DNA, epigenetic methylation, colorectal cancer, cancer biomarkers

## Abstract

Circulating tumour DNA biomarkers are an expanding field in oncology research that offer great potential but are currently often limited in value by overall cost. The aim of this study was to evaluate the efficacy of a novel multi-gene methylation blood test for the identification of colorectal cancer and throughout the spectrum of colorectal disease. Participants were recruited either prior to resection for known CRC or prior to screening colonoscopy after a positive faecal immunochemical test. Blood was collected from participants prior to their procedure being performed. The plasma was separated, and multiplex MethylLight droplet digital PCR was used to analyse for the presence of four methylated genes: *SDC2*, *NPY*, *IKZF1* and *SEPT9*. A total of 537 participants underwent analysis. The *SDC2*/*NPY* genes showed a sensitivity of 33–54% and a specificity of 72–96%, whilst the *IKZF1*/*SEPT9* genes showed a sensitivity of 19–42% and a specificity of 88–96%. Combining the two tests did not significantly increase the test accuracy. The sensitivity for advanced adenoma was 2–15%. There was a significant difference in the frequency of detectable methylation between the participants with CRC and those without CRC. However, neither the sensitivity nor the specificity was superior to current diagnostic screening tests.

## 1. Introduction

Colorectal cancer (CRC) is one of the leading causes of cancer-related mortality worldwide, and screening is an important tool to reduce the burden of disease. Annually, over 900,000 people die from CRC, and over 1.9 million new cases are diagnosed [1]. Historically, the two main forms of diagnostic screening tests used are either invasive (endoscopic) or non-invasive (faecal immunochemical test—FIT). However, there are emerging blood-based tests that may play a role in screening in the future. Screening in Australia is currently carried out using the FIT test and has been shown to reduce the mortality, incidence of advanced-stage CRC and overall incidence of CRC due to the early detection of early stage invasive disease and pre-malignant neoplasia [2,3,4,5]. Despite this proven benefit, there is only a 42% uptake of the screening program in Australia among the eligible population [6].

The poor participation rate has been shown to be partially due to a general preference for blood-based tests rather than faecal-based tests [7]. Additionally, there is evidence that offering a blood-based test may increase the overall screening participation rate compared to the FIT alone [8]. Another issue with FIT is that it is not specifically testing for CRC, and, as a result, there are many other pathologies that can create a false positive result. Hence, there are good reasons to support the search for more precise tests that are more acceptable for the general population. New tests, such as Epi proColon and Colvera, have emerged but have thus far been found to have a limited role in initial screening and diagnostics for CRC [9,10,11].

Aberrant hypermethylation of CpG islands in promotor regions of specific genes has been extensively described as a common epigenetic DNA alteration that is detectable in the early stages of many cancer types [12,13,14,15]. Numerous studies have described hypermethylated genes in CRC that have the potential to be used as diagnostic tests. However, few have made it past the investigation stage and into clinical practice [12,16]. Methylated tumour DNA has been shown to be present in both primary CRC tissue as well as plasma and stool [17]. We have previously described high levels of hypermethylation of 10 genes in CRC tissue that have the potential for use as non-invasive biomarkers of disease [18]. This prospective case–control study aims to investigate the efficacy of a combination of four of these genes for the identification of CRC and for pre-malignant neoplastic lesions.

## 2. Materials and Methods

### 2.1. Patient Recruitment and Ethics

In this multicentre prospective study, patients were recruited, and specimens were collected from three hospitals in the Hunter New England Local Health District between 2017 and 2021. Two cohorts of patients were used in this study. The first cohort consisted of 127 participants who were undergoing surgery for confirmed or suspected CRC. Blood was collected prior to the operation for all participants. The second cohort consists of 457 participants who underwent a screening colonoscopy after having tested positive on the FIT test. Blood was collected after mechanical bowel preparation had been taken by the patient immediately prior to the colonoscopy from all participants. This second cohort includes participants with normal examination findings, as well as those with low-grade adenomas, high-grade adenomas, colorectal cancers, and other gastrointestinal pathologies. This study was approved by the Hunter New England Human Research Ethics Committee (2019/ETH01147, 18/03/21/5.10). Written informed consent for the collection of specimens and further analysis was obtained from all patients prior to their procedures.

### 2.2. Clinical Specimens

Venous blood was collected in up to three 9 mL vacuette tubes from participants immediately prior to the procedure being performed. The samples were processed within 4 h of collection if collected in EDTA tubes and within 24 h if collected in STRECK BCT tubes (Streck, La Vista, NE, USA). The tubes were centrifuged at 2000× *g* for 10 min, plasma was pipetted into a new tube, and this was centrifuged again at 2000× *g* for 10 min. The separated plasma was then aliquoted into separate tubes and immediately stored at −80 °C. Complete histopathological examination and status of the colonoscopy biopsies or surgical specimen were confirmed by a certified pathologist, and, if CRC was detected, they were staged using the TNM system defined by the Union for International Cancer Control (UICC) [19].

### 2.3. DNA Isolation and Bisulfite Treatment

Circulating tumour DNA (ctDNA) was isolated from the plasma specimens using Zymo Quick-cfDNA Serum and Plasma kit (Zymo Research, Irvine, CA, USA). The total cell-free DNA (cfDNA) was quantified using Qubit 2.0, HS-dsDNA assay (Life Technologies, Carlsbad, CA, USA), and stored at −80 °C. Samples were then treated with bisulfite using the EZ DNA Methylation-Gold kit (Zymo Research, Irvine, CA, USA) according to the manufacturer’s instructions for 50 µL input volume and eluted in a volume of 20 µL. Unmethylated and methylated genomic DNA were treated with bisulfite in parallel and used as positive and negative controls. The bisulfite treated cfDNA was stored at −20 °C and was analysed using ML-ddPCR within 48 h of treatment.

### 2.4. MethylLight Droplet Digital PCR (ML-ddPCR) Protocol

ML-ddPCR was performed using the Bio-rad QX200 system (Bio-Rad Laboratories, South Granville, NSW, Australia). Custom primer and probe sequences were designed for the bisulfite-converted methylated alleles of each gene of interest and the Actin-β (ACTB) reference gene (Table 1). The target and reference genes have been previously validated by prior research from this group [18]. All variable gene combinations were assessed statistically in the previous study protocol, and a large number of gene combinations with very similar efficacy were then selected for further optimisation. Alteration of the original primer and probe sequences, as well as further optimisation of the ML-ddPCR assay protocol, was performed to improve the resolution between positive and negative droplet clouds after the combination of target genes into one assay. It was found that the combination of 3 or 4 genes in one PCR well led to reduced resolution of the droplet clouds for ddPCR and hindered the accuracy of the PCR. Duplex reactions were found to be best, and, similarly, numerous combinations of genes were tested, with the most favourable of these being selected for the further primer and probe optimisation that was performed. The four final genes chosen were highly sensitive and specific for CRC in the prior study utilising tissue samples, and, additionally, their combination into two separate duplex ddPCR reactions maintained reproducibly accurate results with minimal interference.

ML-ddPCR was performed using 6 µL volume of bisulfite-converted sample DNA in each reaction well. Stock solutions of target and reference gene primers and probes were made to provide consistent concentration across ddPCR plates. Master mixes for each PCR plate were made to contain a final concentration of 900 nM for the primers of 2 target genes, 450 nM for ACTB primers and 250 nM for all three probes. Sample and master mix were combined to achieve a total end volume in each PCR well of 22 µL, which contained 6 µL of sample, 5 µL of primer and probe solution and 11 µL of Supermix. The 96-well plate was then sealed, centrifuged at 300 rpm for 5 s, gently vortexed for 30 s and recentrifuged at 300 rpm. The plate-seal was removed, and the plate was then run on the QX200 AutoDG Droplet Digital PCR system, immediately foil heat sealed using the PX1 PCR Plate Sealer and run on the Veriti Thermal Cycler. The PCR cycling conditions were 95 °C for 10 min followed by 40 cycles of 94 °C for 30 s, 52 °C for 120 s and finally 98 °C for 10 min and 12 °C holding temperature. The plates were left at the holding temperature of 12 °C for at least 8 h. The plate was then placed into the QX200 Droplet Reader for analysis, and the data were analysed using QuantaSoft software, version QX200 (Bio-rad, Hercules, CA, USA). 

### 2.5. Calculation of the LoD and LoB

Calculation of the limit of blank (LoB) and limit of detection (LoD) was performed in the manner previously reported by this group [18]. However, in this case, the bisulfite-treated unmethylated control (UMC) DNA wells were used instead of the no template control (NTC) wells for the calculation. This was performed to account for the possible background DNA producing a false positive signal. Additionally, a conservative value of 5 times the standard deviation was used to reduce potential false positives in the equation:

LoD = LoB + 5 × (SD_blank_)

The LoD using this method was 5 droplets for the *SDC2*/*NPY* assay, 2 droplets for the *IKZF1*/*SEPT9* assay and 5 droplets for the merged data. The ML-ddPCR assays are quantitative, and there is no clear value of ctDNA fractional abundance that should be used to qualify a positive result. Therefore, alternate cut-offs of more than 1%, 0.5% and 0.25% ctDNA fractional abundance were also used in separate analyses (Supplementary Material: Tables S1–S9).

### 2.6. Statistical Analysis

Statistical analysis was performed using SPSS (v28). Intergroup differences in demographics were measured using Fishers’ exact test (2-tailed, significance *p* < 0.05). Differences in total cfDNA levels were analysed with Mann–Whitney U and Kruskal–Wallis H tests (significance *p* < 0.05). PCR assay results were analysed as both singular tests for *SDC2*/*NPY* (model 1a) or *IKZF1*/*SEPT9* (model 1b), as well as combined tests. The combination of assays was performed in 3 ways; model 2 merged the data for a single test result value; model 3 required only 1 of the assays to be positive; model 4 required both assays to be positive. Test sensitivity and specificity were calculated using crosstabulations, and the association of variables was analysed using Goodman and Kruskal tau (significance *p* < 0.05). Associations with cancer-specific variables, such as T-stage, nodal status, lymphovascular invasion (LVI), site of tumour and pathological stage, were performed using the same method. There were insufficient participants with metastatic disease to analyse possible associations. Multivariate analysis using the Cochran–Mantel–Haenszel (CMH) test was performed for confounding variables such as age, gender, body mass index (BMI), co-morbidities using the Charlson Co-morbidity Index (CCI), immunosuppression, aspirin use and smoking status.

## 3. Results

### 3.1. Population

**Figure 1:**
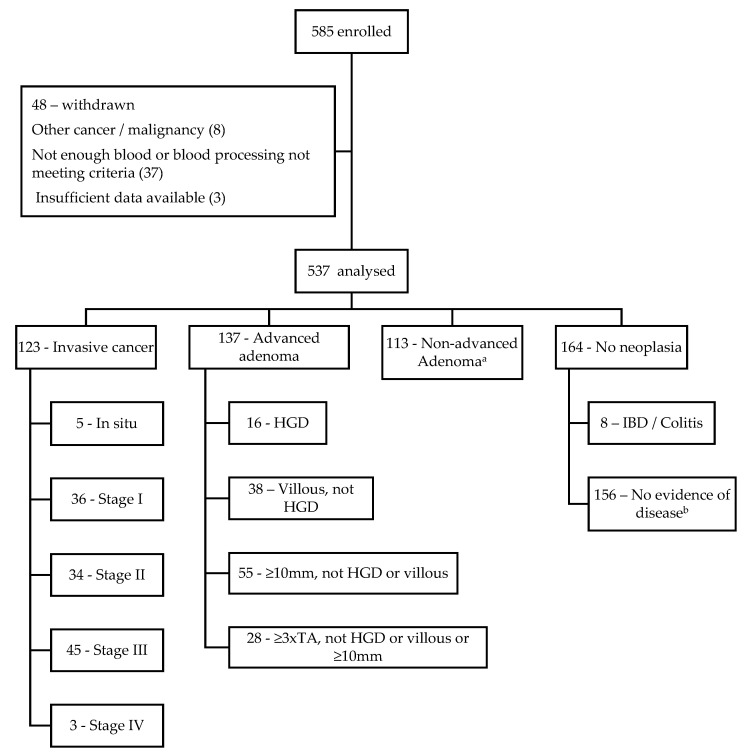
Flow chart of patient recruitment, exclusions and diagnostic categories. HGD, high-grade dysplasia; TA, tubular adenoma; IBD, inflammatory bowel disease; Colitis, non-IBD related colitis causes; NED, no evidence of disease; ^a^ includes all other polyps that do not come under the ‘advanced adenoma’ definition; ^b^ includes hyperplastic polyps, diverticulosis, angiodysplasia and haemorrhoids but excludes cancers, adenomas and inflammations of any kind.

Figure 1 summarises the recruitment of participants, the reasons for exclusion and the number of participants in each diagnostic category. Methylated ctDNA testing was completed on 537 participants, and their demographic characteristics, clinical findings and cfDNA results are shown in Table 2. Proportionally, more participants were aged >70 in the cancer group (*p* < 0001), and a higher rate of smoking was found in the non-cancer group (*p* < 0.05). The CCI score was higher in those patients with cancer (*p* < 0.001), and this remained significant after adjusting for the fact that a solid tumour adds 2 to the CCI score. These three demographic characteristics were also found to be directly associated with the test results (*p* < 0.05). There was no significant difference between groups among all other demographic characteristics. Histopathological features of the patients with CRC are shown in Table 3. 

### 3.2. Sensitivity and Specificity for CRC Using ctDNA

Among the 123 participants with CRC, there were 45 (37%) positive for *SDC2*/*NPY* methylation with a specificity of 96% among the non-cancer group using the LoD method (Table 4). A total of 51 (42%) participants with CRC were positive for *IKZF1*/*SEPT9* methylation with a specificity of 88%. Merging the two assay results (model 2) led to a slightly improved sensitivity (44%) and a specificity within the same range (92%). Model 3 increased the sensitivity at the expense of the specificity, whilst requiring both assays to be positive (model 4) produced the opposite result. Analysis using the cut-off of 1% fractional abundance of methylated alleles resulted in a slight overall increase in specificity with lower sensitivity, whilst a cut-off of 0.25% produced a minor increase in sensitivity at the cost of specificity (Supplementary Materials: Tables S1–S3). There was a variable level of association between the stage of disease and the frequency of positive results across all the models and cut-offs (Table 5, Supplementary Materials: Tables S4–S9). 

### 3.3. Sensitivity for Detection of Adenomas and Colitis

Among the 137 participants with AA, there was a 2% sensitivity with *SDC2*/*NPY* methylation and a 14% sensitivity with *IKZF1*/*SEPT9* methylation (Table 6). Among the 113 participants with NAA, there was a 7% sensitivity with *SDC2*/*NPY* methylation and a 11% sensitivity with *IKZF1*/*SEPT9* methylation. Unsurprisingly requiring only one of the two assays to be positive resulted in the highest sensitivity for both AA and NAA (15%). Although the number of participants with IBD or colitis was small, there was only one positive result from the *IKZF1/SEPT9* assay (13%). 

### 3.4. Association of Cancer-Specific Variables and Confounding Factors

A variable association was seen between assay results and nodal disease. A significant relationship was found between nodal status and both *SDC2*/*NPY* and model 2 results, whilst there was no association between *IKZF1*/*SEPT9* or models 3 or 4 (Supplementary Materials: Section S2). There was no association found for LVI with the single exception of SDC2/NPY 0.25% fractional abundance (*p* = 0.011). There was no association between T-stage or cancer site. Potential demographic and cancer-specific confounding variables produced no change in the significant associations found between test results and the presence of cancer. Despite the associations found with age, CCI and smoking, there remained a significant association after multivariate analysis between cancer and the test results in all models.

## 4. Discussion

This prospective study was undertaken because initial results from a panel of hypermethylated genes in tumour tissue and normal adjacent colonic tissue (NACT) appeared effective when discerning between CRC and NACT [18]. The current study aimed to translate a specific set of these biomarkers into a blood-based test and assess their value. For this purpose, a panel of four genes rather than any singular gene was used for two reasons. Firstly, the panel of genes was thought more likely to capture the heterogeneity of CRC among patients and, therefore, have a higher rate of detection. But most importantly, our previous study had found that the combined panel seemed more effective at differentiating CRC tissue from NACT.

The *SDC2*/*NPY* test showed a sensitivity range of 33–54% and a specificity range of 72–96% for the detection of CRC, whilst the *IKZF1*/*SEPT9* test showed a sensitivity range of 19–42% and a specificity range of 88–96%. Combining the two tests made no significant improvement to these figures across all methods employed. Similarly, there was no improvement when altering the method of cut-off to fractional abundance. In addition to this, there was a low sensitivity for the detection of advanced adenomas (2–15%). The reported sensitivity of FIT for advanced adenomas is reported to be in the range of 20–40% [20]. The relatively low sensitivity for CRC contrasts with this group’s previous findings on tissue samples, and, therefore, FIT remains a comparatively superior screening test. 

The association of increased age with methylation levels is not entirely unexpected. This group’s pilot study on tissue samples found a significant association between age and methylation of the *SDC2* and *IKZF1* genes [18]. Additionally, there is evidence that CpG islands continue to gain methylation as people age [21]. These findings make the clinical applicability of DNA methylation-based tests more difficult. However, it should be noted that there is a similar decrease in FIT sensitivity as age increases and yet it is still an effective screening test [22]. Smoking was found to be inversely associated with both the presence of cancer and the presence of methylation. The inverse association of smoking with methylation has been noted previously with genes such as *F2RL3* and *AHRR* [23]. Finally, there was also an association between the presence of cancer or gene methylation and participant CCI score, which remained significant after adjustment. Although none of these confounders affected the significant association between the assay results and the presence of cancer in this study, it is important to highlight that they must be considered in any future research or clinical applications of epigenetic methylation markers. Furthermore, despite the implications of these associations, it should be noted that FIT and CEA are also associated with the same confounders [22,24,25,26,27]. 

The cohort of patients tested in this study was reasonably large when compared to most of the research in this emerging field. However, the study was limited by the inability to directly compare the efficacy of the genetic markers with FIT due to the whole colonoscopy cohort being FIT positive. A current limitation of ctDNA biomarkers that is illustrated in this study is the ill-defined threshold for a positive test result. The small mass of circulating cell-free DNA that is isolated from plasma is likely to be contaminated by cell-free DNA from sources other than the tumour. Inappropriate handling can lead to large quantities of fragmented DNA released from white blood cell lysis and this can lead to significant dilution of ctDNA. Typically, the ctDNA is only a fraction of the total cell-free DNA isolated, so this can lead to difficulty ascertaining measurable signals and defining appropriate thresholds.

## 5. Conclusions

This study utilised a novel method of ML-ddPCR to investigate the efficacy of a combined four-gene epigenetic methylation biomarker for the identification of CRC. Whilst the results showed a significant difference between participants with CRC and without CRC, the level of sensitivity for detection was not superior to the current diagnostic tests. Additionally, there was a low rate of detection of high-risk advanced adenomas that are currently detected at higher rates using FIT.

## Figures and Tables

**Table 1:** Gene, type and sequence.

Target Gene		
***ACTB***	Forward	TGGTGATGGAGGAGGTTTAGTAAG
	Reverse	ACCAATAAAACCTACTCCTCCCTTA
	Probe	VIC/ACCACCACCCAACACACAATAACAAACA/MGBNFQ
***IKZF1***	Forward	TGCGCGTTTCGTTTTTTGTATCG
	Reverse	GATCCCTACTCGACCTACCCCGC
	Probe	FAM/CGACCGCCTCCCGAATCGC/MGBNFQ
***NPY***	Forward	+C+G+AGGTTTTTTTTGTCGC
	Reverse	ATAC+T+A+T+CGAACGAACG
	Probe	FAM/CAAAAAACGA+A+T+C+G+C+GACAA/3IABkFQ
***SDC2***	Forward	AAATTA+A+T+A+AGTGAGAGGGCGTC
	Reverse	GAC+T+C+A+AACTCGAAAACTCG
	Probe	FAM/CGTAGGAGGAGGAAG+C+G+A+G+C/3IABkFQ
***SEPT9***	Forward	+C+G+T+CGTTGTTTTTCG
	Reverse	CCCACCTTCGAAATCCG
	Probe	FAM/CGTTAACCGCGAAATCCG/MGBNFQ

‘+’ represents Linked Nucleic Acid (LNA) inclusion positions in the primers and probes.

**Table 2:** Demographic and clinical findings.

	No. Cases	Age (Years)	Gender		BMI	CCI	NSAID Use		Immunosuppression		Smoking Status			cfDNA	
	n (%)	Median (Min-Max)	Women	Men	Median (Min-Max)	Median (Min-Max)	No	Yes	No	Yes	Non-Smoker	Ex-Smoker	Smoker	Median (Range)	Mean (SD)
			n (%)				n (%)		n (%)		n (%)				
**Cancer**	123 (23)	70 (37–92)	47 (38)	76 (62)	28 (20–54)	5 (2–9)	95 (77)	28 (23)	115 (93)	8 (7)	58 (47)	53 (43)	12 (10)	6.17 (56.1)	7.96 (7.02)
**Stage 0**	5	65 (58–86)	2 (40)	3 (60)	26 (21–32)	4 (4–7)	3 (60)	2 (40)	5 (100)	0	2 (40)	3 (60)	0	4.37 (6.9)	5.21 (2.73)
**Stage I**	36	68 (37–85)	17 (47)	19 (53)	27 (20–49)	5 (2–9)	29 (81)	7 (19)	34 (94)	2 (6)	16 (44)	16 (44)	4 (11)	6.45 (14.6)	6.32 (3.24)
**Stage II**	34	72 (48–91)	12 (35)	22 (65)	30 (20–46)	5 (2–9)	24 (71)	10 (29)	32 (94)	2 (6)	18 (53)	10 (29)	6 (18)	6.90 (54.7)	9.0 (9.14)
**Stage III**	45	71 (48–92)	15 (33)	30 (67)	31 (20–54)	5 (2–9)	36 (80)	9 (20)	41 (91)	4 (9)	20 (44)	24 (53)	1 (2)	6.56 (37.6)	8.43 (7.16)
**Stage IV**	3	69 (56–87)	1 (33)	2 (67)	29 (25–29)	8 (3–8)	3 (100)	0	3 (100)	0	2 (67)	0	1 (33)	6.0 (22.4)	13.35 (12.89)
**AA**	137 (25)	64 (47–85)	48 (35)	89 (65)	29 (19–43)	3 (0–9)	112 (82)	25 (18)	135 (98)	2 (2)	62 (45)	42 (31)	33 (24)	6.07 (32.5)	7.04 (4.42)
**NAA**	113 (21)	64 (49–79)	57 (50)	56 (50)	29 (19–41)	2 (0–8)	101 (89)	12 (11)	109 (96)	4 (4)	50 (44)	34 (30)	29 (26)	5.83 (21.2)	6.65 (3.55)
**No neoplasia ^a^**	164 (31)	62 (45–77)	91 (55)	73 (45)	28 (17–42)	2 (0–6)	137 (83)	27 (17)	157 (96)	7 (4)	80 (49)	56 (34)	28 (17)	5.74 (21.9)	6.34 (2.97)
**IBD/Colitis**	8	58 (47–69)	6 (75)	2 (25)	24 (20–36)	1 (0–3)	8 (100)	0	8 (100)	0	3 (38)	5 (62)	0	5.0 (5.1)	4.83 (1.62)
**NED ^b^**	156	62 (45–77)	85 (54)	71 (46)	28 (17–42)	2 (0–6)	129 (83)	27 (17)	149 (95)	7 (5)	77 (49)	51 (33)	28 (18)	5.84 (21.9)	6.41 (3.0)
**Study Cohort Overall**	537 (100)	64 (37–92)	243 (45)	294 (55)	29 (17–54)	3 (0–9)	445 (83)	92 (17)	516 (96)	21 (4)	250 (47)	185 (34)	102 (19)	6.02 (56.4)	6.95 (4.68)
***p*-value ^c^**		<0.001	0.08		0.885	<0.001	0.076		0.11		<0.05			0.154	

BMI, body mass index; CCI, Charlson comorbidity Index; AA, advanced adenoma; NAA, non-advanced adenoma; NED, no evidence of disease; ^a^ all cases without neoplasia, i.e., excluding cases with cancer or adenoma; ^b^ includes hyperplastic polyps, diverticulosis, angiodysplasia and haemorrhoids but excludes cancers, adenomas and inflammations of any kind; ^c^ value based on entire cohort with respect to cancer vs. no cancer.

**Table 3:** Histopathological features of CRC patients.

		Cancer n (%)
**T-Stage**	In Situ	5 (4)
	T1	10 (8)
	T2	33 (27)
	T3	61 (50)
	T4	14 (11)
**Nodal status**	Negative	75 (61)
	Positive	48 (39)
**Metastatic disease**	Negative	120 (98)
	Positive	3 (2)
**LVI**	Negative	84 (68)
	Positive	39 (32)
**Site of Cancer**	Right	49 (40)
	Left	73 (59)
	Synchronous	1 (1)
**Total**		123

LVI, Lymphovascular invasion.

**Table 4:** Sensitivity and specificity for the detection of cancer (LoD).

Analysis Method (LoD*)*	Status	Cancer		Total	*p*-Value
		No Cancer	Cancer		
		n (%)			
**Model 1a**	Negative	396 (96)	78 (63)	474	<0.001
	Positive	18 (4)	45 (37)	63	
**Model 1b**	Negative	366 (88)	72 (58)	438	<0.001
	Positive	48 (12)	51 (42)	99	
**Model 2**	Negative	382 (92)	69 (56)	451	<0.001
	Positive	32 (8)	54 (44)	86	
**Model 3**	Negative	353 (85)	61 (50)	414	<0.001
	Positive	61 (15)	62 (50)	123	
**Model 4**	Negative	409 (99)	89 (72)	498	<0.001
	Positive	5 (1)	34 (28)	39	
	Total	414	123	537	

**Table 5:** Sensitivity for detection based on stage of cancer (LoD).

Analysis Method (LoD)	Status	Stage					Total	*p*-Value
		In Situ	1	2	3	4		
		N (%)						
**Model 1a**	Negative	5 (100)	25 (69)	24 (71)	23 (51)	1 (33)	78	0.074
	Positive	0	11 (31)	10 (29)	22 (49)	2 (67)	45	
**Model 1b**	Negative	4 (80)	20 (56)	21 (62)	26 (58)	1 (33)	72	0.749
	Positive	1 (20)	16 (44)	13 (38)	19 (42)	2 (67)	51	
**Model 2**	Negative	5 (100)	21 (58)	22 (65)	21 (47)	0	69	0.026
	Positive	0	15 (42)	12 (35)	24 (53)	3 (100)	54	
**Model 3**	Negative	4 (80)	18 (50)	19 (56)	19 (42)	1 (33)	61	0.469
	Positive	1 (20)	18 (50)	15 (44)	26 (58)	2 (67)	62	
**Model 4**	Negative	5 (100)	27 (75)	26 (76)	30 (67)	1 (33)	89	0.244
	Positive	0	9 (25)	8 (24)	15 (33)	2 (67)	34	
	Total	5	36	34	45	3	123	

**Table 6:** Sensitivity for detection based on principle diagnosis (LoD).

Analysis Method (LoD)	Status	Principle Diagnosis					Total	*p*-Value
		Cancer	AA	NAA	IBD/Colitis	NED ^a^		
		N (%)						
**Model 1a**	Negative	78 (63)	134 (98)	105 (93)	8 (100)	149 (95)	474	<0.001
	Positive	45 (37)	3 (2)	8 (7)	0	7 (5)	63	
**Model 1b**	Negative	72 (58)	118 (86)	101 (89)	7 (87)	140 (90)	438	<0.001
	Positive	51 (42)	19 (14)	12 (11)	1 (13)	16 (10)	99	
**Model 2**	Negative	69 (56)	128 (93)	102 (90)	8 (100)	144 (92)	451	<0.001
	Positive	54 (44)	9 (7)	11 (10)	0	12 (8)	86	
**Model 3**	Negative	61 (50)	117 (85)	96 (85)	7 (87)	133 (85)	414	<0.001
	Positive	62 (50)	20 (15)	17 (15)	1 (13)	23 (15)	123	
**Model 4**	Negative	89 (72)	135 (98)	110 (97)	8 (100)	156 (100)	498	<0.001
	Positive	34 (28)	2 (2)	3 (3)	0	0	39	
	Total	123	137	113	8	156	537	

AA, advanced adenoma; NAA, non-advanced adenoma; IBD, inflammatory bowel disease; Colitis, non-IBD related colitis causes; NED, no evidence of disease; ^a^ includes hyperplastic polyps, diverticulosis, angiodysplasia and haemorrhoids but excludes cancers, adenomas and inflammations of any kind.

## Data Availability

Part of the data presented in this study are available within the article and Supplementary Material S1. The rest of the data presented in this study are available on request to the corresponding author. The data are not publicly available due to ethics approval restrictions and privacy reasons for the participants in the study.

## Supplementary Materials

The following supporting information can be downloaded at: https://www.mdpi.com/article/10.3390/medsci11030060/s1. Supplementary Material S1. Section S1: Fractional Abundance Tables. Section S2: Cancer Specific confounding variables.
