# Supplementary material for: Evaluation of a Multi-Gene Methylation Blood-Test for the Detection of Colorectal Cancer
Source: Med Sci (Basel). 2023 Sep 15;11(3):60. doi: 10.3390/medsci11030060 (PMC10534804; doi:10.3390/medsci11030060)
Supplement: Supplementary file 1 [file medsci-11-00060-s001.zip › medsci-2578087-supplementary.pdf]

Supplementary Materials 1

Section 1: Fractional Abundance Tables

Table 1: Sensitivity and specificity for detection of cancer (1% Fr.Ab.)

Analysis Method (1% Fr.Ab.)	Status	Cancer		Total	P-Value
		No Cancer	Cancer		
		N (%)			
Model 1a	Negative	375 (91)	82 (67)	457	<0.001
	Positive	39 (9)	41 (33)	80	
Model 1b	Negative	397 (96)	100 (81)	497	<0.001
	Positive	17 (4)	23 (19)	40	
Model 2	Negative	385 (93)	89 (72)	474	<0.001
	Positive	29 (7)	34 (28)	63	
Model 3	Negative	366 (88)	78 (63)	444	<0.001
	Positive	48 (12)	45 (37)	93	
Model 4	Negative	406 (98)	104 (85)	510	<0.001
	Positive	8 (2)	19 (15)	27	
	Total	414	123	537	

Fr.Ab, Fractional Abundance

Table 2: Sensitivity and specificity for detection of cancer (0.5% Fr.Ab.)

Analysis Method (0.5% Fr.Ab.)	Status	Cancer		Total	P-Value
		No Cancer	Cancer		
		N (%)			
Model 1a	Negative	342 (83)	71 (58)	413	<0.001
	Positive	72 (17)	52 (42)	124	
Model 1b	Negative	386 (93)	90 (73)	476	<0.001
	Positive	28 (7)	33 (27)	61	
Model 2	Negative	359 (87)	74 (60)	433	<0.001
	Positive	55 (13)	49 (40)	104	
Model 3	Negative	329 (79)	66 (54)	395	<0.001
	Positive	85 (21)	57 (46)	142	
Model 4	Negative	399 (96)	95 (77)	494	<0.001
	Positive	15 (4)	28 (23)	43	
	Total	414	123	537	

Fr.Ab, Fractional Abundance

Table 3: Sensitivity and specificity for detection of cancer (0.25% Fr.Ab.)

Analysis Method (0.25% Fr.Ab.)	Status	Cancer		Total	P-Value
		No Cancer	Cancer		
		N (%)			
Model 1a	Negative	297 (72)	56 (46)	353	<0.001
	Positive	117 (28)	67 (54)	184	
Model 1b	Negative	369 (89)	79 (64)	448	<0.001
	Positive	45 (11)	44 (36)	89	
Model 2	Negative	324 (78)	61 (50)	385	<0.001
	Positive	90 (22)	62 (50)	152	
Model 3	Negative	271 (65)	46 (37)	317	<0.001
	Positive	143 (35)	77 (63)	220	
Model 4	Negative	395 (95)	89 (72)	484	<0.001
	Positive	19 (5)	34 (28)	53	
	Total	414	123	537	

Fr.Ab, Fractional Abundance

Table 4: Sensitivity for detection based on stage of cancer (1% Fr.Ab.)

Analysis Method (1% Fr.Ab.)	Status	Stage					Total	P-Value
		0	1	2	3	4		
		N (%)						
Model 1a	Negative	4 (80)	28 (78)	24 (71)	26 (58)	0	82	<0.05
	Positive	1 (20)	8 (22)	10 (29)	19 (42)	3 (100)	41	
Model 1b	Negative	5 (100)	29 (81)	27 (79)	38 (84)	1 (33)	100	0.199
	Positive	0	7 (19)	7 (21)	7 (16)	2 (67)	23	
Model 2	Negative	4 (80)	27 (75)	26 (77)	32 (71)	0	89	0.076
	Positive	1 (20)	9 (25)	8 (23)	13 (29)	3 (100)	34	
Model 3	Negative	4 (80)	24 (67)	24 (71)	26 (58)	0	78	0.115
	Positive	1 (20)	12 (33)	10 (29)	19 (42)	3 (100)	45	
Model 4	Negative	5 (100)	33 (92)	27 (79)	38 (84)	1 (33)		0.067
	Positive	0	3 (8)	7 (21)	7 (16)	2 (67)		
	Total	5	36	34	45	3	123	

Fr.Ab, Fractional Abundance

Table 5: Sensitivity for detection based on stage of cancer (Fr.Ab. 0.5%)

Analysis Method (0.5% Fr.Ab.)	Status	Stage					Total	P-Value
		0	1	2	3	4		
		N (%)						
Model 1a	Negative	2 (40)	24 (67)	23 (68)	22 (49)	0	71	0.06
	Positive	3 (60)	12 (33)	11 (32)	23 (51)	3 (100)	52	
Model 1b	Negative	4 (80)	26 (72)	26 (77)	34 (76)	0	90	0.071
	Positive	1 (20)	10 (28)	8 (23)	11 (24)	3 (100)	33	
Model 2	Negative	3 (60)	24 (67)	24 (71)	23 (51)	0	74	0.075
	Positive	2 (40)	12 (33)	10 (29)	22 (49)	3 (100)	49	
Model 3	Negative	2 (40)	21 (58)	22 (65)	21 (47)	0	66	0.15
	Positive	3 (60)	15 (42)	12 (35)	24 (53)	3 (100)	57	
Model 4	Negative	4 (80)	29 (81)	27 (79)	35 (78)	0	95	0.036
	Positive	1 (20)	7 (19)	7 (21)	10 (22)	3 (100)	28	
	Total	5	36	34	45	3	123	

Fr.Ab, Fractional Abundance

Table 6: Sensitivity for detection based on stage of cancer (Fr.Ab. 0.25%)

Analysis Method (0.25% Fr.Ab.)	Status	Stage					Total	P-Value
		0	1	2	3	4		
		N (%)						
Model 1a	Negative	1 (20)	19 (53)	21 (62)	15 (33)	0	56	0.021
	Positive	4 (80)	17 (47)	13 (38)	30 (67)	3 (100)	67	
Model 1b	Negative	4 (80)	22 (61)	24 (71)	29 (64)	0	79	0.15
	Positive	1 (20)	14 (39)	10 (29)	16 (36)	3 (100)	44	
Model 2	Negative	2 (40)	19 (53)	22 (65)	18 (40)	0	61	0.082
	Positive	3 (60)	17 (47)	12 (35)	27 (60)	3 (100)	62	
Model 3	Negative	1 (20)	14 (39)	18 (53)	13 (29)	0	46	0.109
	Positive	4 (80)	22 (61)	16 (47)	32 (71)	3 (100)	77	
Model 4	Negative	4 (80)	27 (75)	27 (79)	31 (69)	0	89	0.055
	Positive	1 (20)	9 (25)	7 (21)	14 (31)	3 (100)	34	
	Total	5	36	34	45	3	123	

Fr.Ab, Fractional Abundance

Table 7: Sensitivity for detection based on principle diagnosis (1% Fr.Ab.)

Analysis Method (1% Fr.Ab.)	Status	Principle Diagnosis					Total	P-Value
		Cancer	AA	NAA	IBD/Colitis	NED		
		N (%)						
Model 1a	Negative	82 (67)	128 (93)	102 (90)	7 (87)	138 (88)	457	<0.001
	Positive	41 (33)	9 (7)	11 (10)	1 (13)	18 (12)	80	
Model 1b	Negative	100 (81)	128 (93)	109 (96)	8 (100)	152 (97)	497	<0.001
	Positive	23 (19)	9 (7)	4 (4)	0	4 (3)	40	
Model 2	Negative	89 (72)	125 (91)	107 (95)	8 (100)	145 (93)	474	<0.001
	Positive	34 (28)	12 (9)	6 (5)	0	11 (7)	63	
Model 3	Negative	78 (63)	123 (90)	101 (89)	7 (87)	135 (86)	444	<0.001
	Positive	45 (37)	14 (10)	12 (11)	1 (13)	21 (14)	93	
Model 4	Negative	104 (85)	133 (97)	110 (97)	8 (100)	155 (99)	510	<0.001
	Positive	19 (15)	4 (3)	3 (3)	0	1 (1)	27	
	Total	123	137	113	8	156	537	

Fr.Ab, Fractional Abundance; AA, advanced adenoma; NAA, non-advanced adenoma; IBD, inflammatory bowel disease; Colitis, non-IBD related colitis causes; NED, no evidence of disease; [a]includes hyperplastic polyps, diverticulosis, angiodysplasia and haemorrhoids but excludes cancer, adenomas and inflammation of any kind.

Table 8: Sensitivity for detection based on principle diagnosis (0.5% Fr.Ab.)

Analysis Method (0.5% Fr.Ab.)	Status	Principle Diagnosis					Total	P-Value
		Cancer	AA	NAA	IBD/Colitis	NED		
		N (%)						
Model 1a	Negative	71 (58)	112 (82)	97 (86)	7 (87)	126 (81)	413	<0.001
	Positive	52 (42)	25 (18)	16 (14)	1(13)	30 (19)	124	
Model 1b	Negative	90 (73)	123 (90)	108 (96)	8 (100)	147 (94)	476	<0.001
	Positive	33 (27)	14 (10)	5 (4)	0	9 (6)	61	
Model 2	Negative	74 (60)	121 (88)	99 (88)	7 (87)	132 (85)	433	<0.001
	Positive	49 (40)	16 (12)	14 (12)	1(13)	24 (15)	104	
Model 3	Negative	66 (54)	104 (76)	96 (85)	7 (87)	122 (78)	395	<0.001
	Positive	57 (46)	33 (24)	17 (15)	1(13)	34 (22)	142	
Model 4	Negative	95 (77)	131 (96)	109 (96)	8 (100)	151 (97)	494	<0.001
	Positive	28 (23)	6 (4)	4 (4)	0	5 (3)	43	
	Total	123	137	113	8	156	537	

Fr.Ab, Fractional Abundance; AA, advanced adenoma; NAA, non-advanced adenoma; IBD, inflammatory bowel disease; Colitis, non-IBD related colitis causes; NED, no evidence of disease; [a]includes hyperplastic polyps, diverticulosis, angiodysplasia and haemorrhoids but excludes cancer, adenomas and inflammation of any kind.

Table 9: Sensitivity for detection based on principle diagnosis (0.25% Fr.Ab.)

Analysis Method (0.25% Fr.Ab.)	Status	Principle Diagnosis					Total	P-Value
		Cancer	AA	NAA	IBD/Colitis	NED		
		N (%)						
Model 1a	Negative	56 (46)	96 (70)	82 (73)	6 (75)	113 (72)	353	<0.001
	Positive	67 (55)	41 (30)	31 (27)	2 (25)	43 (28)	184	
Model 1b	Negative	79 (64)	115 (84)	103 (91)	8 (100)	143 (92)	448	<0.001
	Positive	44 (36)	22 (16)	10 (9)	0	13 (8)	89	
Model 2	Negative	61 (50)	103 (75)	95 (84)	7 (87)	119 (76)	385	<0.001
	Positive	62 (50)	34 (25)	18 (16)	1 (13)	37 (24)	152	
Model 3	Negative	46 (37)	81 (59)	77 (68)	6 (75)	107 (69)	317	<0.001
	Positive	77 (63)	56 (41)	36 (32)	2 (25)	49 (31)	220	
Model 4	Negative	89 (72)	130 (95)	108 (96)	8 (100)	149 (95)	484	<0.001
	Positive	34 (28)	7 (5)	5 (4)	0	7 (5)	53	
	Total	123	137	113	8	156	537	

Fr.Ab, Fractional Abundance; AA, advanced adenoma; NAA, non-advanced adenoma; IBD, inflammatory bowel disease; Colitis, non-IBD related colitis causes; NED, no evidence of disease; [a]includes hyperplastic polyps, diverticulosis, angiodysplasia and haemorrhoids but excludes cancer, adenomas and inflammation of any kind.

Section 2: Cancer specific confounding variables

T-Stage * SDC_NPY_LOD_D1

Crosstab

			SDC_NPY_LOD_D1		
			Negative	Positive	Total
T_Stage	1	Count	5	0	5
		% within T_Stage	100.0%	0.0%	100.0%
	2	Count	5	5	10
		% within T_Stage	50.0%	50.0%	100.0%
	3	Count	23	10	33
		% within T_Stage	69.7%	30.3%	100.0%
	4	Count	37	24	61
		% within T_Stage	60.7%	39.3%	100.0%
	5	Count	8	6	14
		% within T_Stage	57.1%	42.9%	100.0%
Total		Count	78	45	123
		% within T_Stage	63.4%	36.6%	100.0%

Directional Measures

			Value	Asymptotic Standard Error[a]	Approximate T	Approximate Significance	Exact Significance
Nominal by Nominal	Lambda	Symmetric	.000	.000	.[b]	.[b]	
		T_Stage Dependent	.000	.000	.[b]	.[b]	
		SDC_NPY_LOD_D1 Dependent	.000	.000	.[b]	.[b]	
	Goodman and Kruskal tau	T_Stage Dependent	.006	.007		.601[c]	.547
		SDC_NPY_LOD_D1 Dependent	.038	.021		.328[c]	.339

a. Not assuming the null hypothesis.

b. Cannot be computed because the asymptotic standard error equals zero.

c. Based on chi-square approximation

T-Stage * IKZF_SEPT9_LOD_D1

Crosstab

			IKZF_SEPT9_LOD_D1		
			Negative	Positive	Total
T_Stage	1	Count	4	1	5
		% within T_Stage	80.0%	20.0%	100.0%
	2	Count	6	4	10
		% within T_Stage	60.0%	40.0%	100.0%
	3	Count	19	14	33
		% within T_Stage	57.6%	42.4%	100.0%
	4	Count	35	26	61
		% within T_Stage	57.4%	42.6%	100.0%
	5	Count	8	6	14
		% within T_Stage	57.1%	42.9%	100.0%
Total		Count	72	51	123
		% within T_Stage	58.5%	41.5%	100.0%

Directional Measures

			Value	Asymptotic Standard Error[a]	Approximate T	Approximate Significance	Exact Significance
Nominal by Nominal	Lambda	Symmetric	.000	.000	.[b]	.[b]	
		T_Stage Dependent	.000	.000	.[b]	.[b]	
		IKZF_SEPT9_LOD_D1 Dependent	.000	.000	.[b]	.[b]	
	Goodman and Kruskal tau	T_Stage Dependent	.001	.002		.985[c]	.979
		IKZF_SEPT9_LOD_D1 Dependent	.008	.014		.909[c]	.920

a. Not assuming the null hypothesis.

b. Cannot be computed because the asymptotic standard error equals zero.

c. Based on chi-square approximation

T-Stage * MERGED_LOD_D1

Crosstab

			MERGED_LOD_D1		
			Negative	Positive	Total
T_Stage	1	Count	5	0	5
		% within T_Stage	100.0%	0.0%	100.0%
	2	Count	5	5	10
		% within T_Stage	50.0%	50.0%	100.0%
	3	Count	19	14	33
		% within T_Stage	57.6%	42.4%	100.0%
	4	Count	32	29	61
		% within T_Stage	52.5%	47.5%	100.0%
	5	Count	8	6	14
		% within T_Stage	57.1%	42.9%	100.0%
Total		Count	69	54	123
		% within T_Stage	56.1%	43.9%	100.0%

Directional Measures

			Value	Asymptotic Standard Error[a]	Approximate T	Approximate Significance	Exact Significance
Nominal by Nominal	Lambda	Symmetric	.000	.000	.[b]	.[b]	
		T_Stage Dependent	.000	.000	.[b]	.[b]	
		MERGED_LOD_D1 Dependent	.000	.000	.[b]	.[b]	
	Goodman and Kruskal tau	T_Stage Dependent	.004	.006		.725[c]	.669
		MERGED_LOD_D1 Dependent	.036	.011		.356[c]	.360

a. Not assuming the null hypothesis.

b. Cannot be computed because the asymptotic standard error equals zero.

c. Based on chi-square approximation

T-Stage * E_O_LOD

Crosstab

			E_O_LOD		
			Negative	Positive	Total
T_Stage	1	Count	4	1	5
		% within T_Stage	80.0%	20.0%	100.0%
	2	Count	4	6	10
		% within T_Stage	40.0%	60.0%	100.0%
	3	Count	16	17	33
		% within T_Stage	48.5%	51.5%	100.0%
	4	Count	29	32	61
		% within T_Stage	47.5%	52.5%	100.0%
	5	Count	8	6	14
		% within T_Stage	57.1%	42.9%	100.0%
Total		Count	61	62	123
		% within T_Stage	49.6%	50.4%	100.0%

Directional Measures

			Value	Asymptotic Standard Error[a]	Approximate T[b]	Approximate Significance	Exact Significance
Nominal by Nominal	Lambda	Symmetric	.041	.034	1.153	.249	
		T_Stage Dependent	.000	.000	.[c]	.[c]	
		E_O_LOD Dependent	.082	.068	1.153	.249	
	Goodman and Kruskal tau	T_Stage Dependent	.002	.004		.881[d]	.849
		E_O_LOD Dependent	.022	.023		.621[d]	.626

a. Not assuming the null hypothesis.

b. Using the asymptotic standard error assuming the null hypothesis.

c. Cannot be computed because the asymptotic standard error equals zero.

d. Based on chi-square approximation

T-Stage * BOTH_LOD

Crosstab

			BOTH_LOD		
			Negative	Positive	Total
T_Stage	1	Count	5	0	5
		% within T_Stage	100.0%	0.0%	100.0%
	2	Count	7	3	10
		% within T_Stage	70.0%	30.0%	100.0%
	3	Count	26	7	33
		% within T_Stage	78.8%	21.2%	100.0%
	4	Count	43	18	61
		% within T_Stage	70.5%	29.5%	100.0%
	5	Count	8	6	14
		% within T_Stage	57.1%	42.9%	100.0%
Total		Count	89	34	123
		% within T_Stage	72.4%	27.6%	100.0%

Directional Measures

			Value	Asymptotic Standard Error[a]	Approximate T	Approximate Significance	Exact Significance
Nominal by Nominal	Lambda	Symmetric	.000	.000	.[b]	.[b]	
		T_Stage Dependent	.000	.000	.[b]	.[b]	
		BOTH_LOD Dependent	.000	.000	.[b]	.[b]	
	Goodman and Kruskal tau	T_Stage Dependent	.006	.007		.558[c]	.519
		BOTH_LOD Dependent	.035	.026		.366[c]	.367

a. Not assuming the null hypothesis.

b. Cannot be computed because the asymptotic standard error equals zero.

c. Based on chi-square approximation

Nodal Status * SDC_NPY_LOD_D1

Crosstab

			SDC_NPY_LOD_D1		
			Negative	Positive	Total
N_Status	Nodes negative	Count	54	21	75
		% within N_Status	72.0%	28.0%	100.0%
	Nodes positive	Count	24	24	48
		% within N_Status	50.0%	50.0%	100.0%
Total		Count	78	45	123
		% within N_Status	63.4%	36.6%	100.0%

Directional Measures

			Value	Asymptotic Standard Error[a]	Approximate T[b]	Approximate Significance	Exact Significance
Nominal by Nominal	Lambda	Symmetric	.032	.071	.448	.654	
		N_Status Dependent	.063	.135	.448	.654	
		SDC_NPY_LOD_D1 Dependent	.000	.000	.[c]	.[c]	
	Goodman and Kruskal tau	N_Status Dependent	.050	.040		.014[d]	.021
		SDC_NPY_LOD_D1 Dependent	.050	.040		.014[d]	.021

a. Not assuming the null hypothesis.

b. Using the asymptotic standard error assuming the null hypothesis.

c. Cannot be computed because the asymptotic standard error equals zero.

d. Based on chi-square approximation

Nodal Status * IKZF_SEPT9_LOD_D1

Crosstab

			IKZF_SEPT9_LOD_D1 Negative	IKZF_SEPT9_LOD_D1 Positive	Total
N_Status	Nodes negative	Count	45	30	75
		% within N_Status	60.0%	40.0%	100.0%
	Nodes positive	Count	27	21	48
		% within N_Status	56.3%	43.8%	100.0%
Total		Count	72	51	123
		% within N_Status	58.5%	41.5%	100.0%

Directional Measures

			Value	Asymptotic Standard Error[a]	Approximate T	Approximate Significance	Exact Significance
Nominal by Nominal	Lambda	Symmetric	.000	.000	.[b]	.[b]	
		N_Status Dependent	.000	.000	.[b]	.[b]	
		IKZF_SEPT9_LOD_D1 Dependent	.000	.000	.[b]	.[b]	
	Goodman and Kruskal tau	N_Status Dependent	.001	.007		.682[c]	.711
		IKZF_SEPT9_LOD_D1 Dependent	.001	.007		.682[c]	.711

a. Not assuming the null hypothesis.

b. Cannot be computed because the asymptotic standard error equals zero.

c. Based on chi-square approximation

Nodal Status * MERGED_LOD_D1

Crosstab

			MERGED_LOD_D1 Negative	MERGED_LOD_D1 Positive	Total
N_Status	Nodes negative	Count	48	27	75
		% within N_Status	64.0%	36.0%	100.0%
	Nodes positive	Count	21	27	48
		% within N_Status	43.8%	56.3%	100.0%
Total		Count	69	54	123
		% within N_Status	56.1%	43.9%	100.0%

Directional Measures

			Value	Asymptotic Standard Error[a]	Approximate T[b]	Approximate Significance	Exact Significance
Nominal by Nominal	Lambda	Symmetric	.059	.066	.869	.385	
		N_Status Dependent	.000	.000	.[c]	.[c]	
		MERGED_LOD_D1 Dependent	.111	.121	.869	.385	
	Goodman and Kruskal tau	N_Status Dependent	.040	.035		.028[d]	.040
		MERGED_LOD_D1 Dependent	.040	.035		.028[d]	.040

a. Not assuming the null hypothesis.

b. Using the asymptotic standard error assuming the null hypothesis.

c. Cannot be computed because the asymptotic standard error equals zero.

d. Based on chi-square approximation

Nodal Status * E_O_LOD

Crosstab

			E_O_LOD		
			Negative	Positive	Total
N_Status	Nodes negative	Count	41	34	75
		% within N_Status	54.7%	45.3%	100.0%
	Nodes positive	Count	20	28	48
		% within N_Status	41.7%	58.3%	100.0%
Total		Count	61	62	123
		% within N_Status	49.6%	50.4%	100.0%

Directional Measures

			Value	Asymptotic Standard Error[a]	Approximate T[b]	Approximate Significance	Exact Significance
Nominal by Nominal	Lambda	Symmetric	.064	.077	.810	.418	
		N_Status Dependent	.000	.000	.[c]	.[c]	
		E_O_LOD Dependent	.115	.134	.810	.418	
	Goodman and Kruskal tau	N_Status Dependent	.016	.023		.161[d]	.197
		E_O_LOD Dependent	.016	.023		.161[d]	.197

a. Not assuming the null hypothesis.

b. Using the asymptotic standard error assuming the null hypothesis.

c. Cannot be computed because the asymptotic standard error equals zero.

d. Based on chi-square approximation

Nodal Status * BOTH_LOD

Crosstab

			BOTH_LOD		
			Negative	Positive	Total
N_Status	Nodes negative	Count	58	17	75
		% within N_Status	77.3%	22.7%	100.0%
	Nodes positive	Count	31	17	48
		% within N_Status	64.6%	35.4%	100.0%
Total		Count	89	34	123
		% within N_Status	72.4%	27.6%	100.0%

Directional Measures

			Value	Asymptotic Standard Error[a]	Approximate T	Approximate Significance	Exact Significance
Nominal by Nominal	Lambda	Symmetric	.000	.000	.[b]	.[b]	
		N_Status Dependent	.000	.000	.[b]	.[b]	
		BOTH_LOD Dependent	.000	.000	.[b]	.[b]	
	Goodman and Kruskal tau	N_Status Dependent	.019	.025		.125[c]	.150
		BOTH_LOD Dependent	.019	.025		.125[c]	.150

a. Not assuming the null hypothesis.

b. Cannot be computed because the asymptotic standard error equals zero.

c. Based on chi-square approximation

LVI_final * SDC_NPY_LOD_D1

Crosstab

			SDC_NPY_LOD_D1 Negative	Positive	Total
LVI_final	0	Count	58	26	84
		% within LVI_final	69.0%	31.0%	100.0%
	1	Count	20	19	39
		% within LVI_final	51.3%	48.7%	100.0%
Total		Count	78	45	123
		% within LVI_final	63.4%	36.6%	100.0%

Directional Measures

			Value	Asymptotic Standard Error[a]	Approximate T	Approximate Significance	Exact Significance
Nominal by Nominal	Lambda	Symmetric	.000	.000	.[b]	.[b]	
		LVI_final Dependent	.000	.000	.[b]	.[b]	
		SDC_NPY_LOD_D1 Dependent	.000	.000	.[b]	.[b]	
	Goodman and Kruskal tau	LVI_final Dependent	.029	.031		.058[c]	.071
		SDC_NPY_LOD_D1 Dependent	.029	.031		.058[c]	.071

a. Not assuming the null hypothesis.

b. Cannot be computed because the asymptotic standard error equals zero.

c. Based on chi-square approximation

LVI_final * IKZF_SEPT9_LOD_D1

Crosstab

			IKZF_SEPT9_LOD_D1 Negative	Positive	Total
LVI_final	0	Count	50	34	84
		% within LVI_final	59.5%	40.5%	100.0%
	1	Count	22	17	39
		% within LVI_final	56.4%	43.6%	100.0%
Total		Count	72	51	123
		% within LVI_final	58.5%	41.5%	100.0%

Directional Measures

			Value	Asymptotic Standard Error[a]	Approximate T	Approximate Significance	Exact Significance
Nominal by Nominal	Lambda	Symmetric	.000	.000	.[b]	.[b]	
		LVI_final Dependent	.000	.000	.[b]	.[b]	
		IKZF_SEPT9_LOD_D1 Dependent	.000	.000	.[b]	.[b]	
	Goodman and Kruskal tau	LVI_final Dependent	.001	.005		.745[c]	.845
		IKZF_SEPT9_LOD_D1 Dependent	.001	.005		.745[c]	.845

a. Not assuming the null hypothesis.

b. Cannot be computed because the asymptotic standard error equals zero.

c. Based on chi-square approximation

LVI_final * MERGED_LOD_D1

Crosstab

			MERGED_LOD_D1		
			Negative	Positive	Total
LVI_final	0	Count	52	32	84
		% within LVI_final	61.9%	38.1%	100.0%
	1	Count	17	22	39
		% within LVI_final	43.6%	56.4%	100.0%
Total		Count	69	54	123
		% within LVI_final	56.1%	43.9%	100.0%

Directional Measures

			Value	Asymptotic Standard Error[a]	Approximate T[b]	Approximate Significance	Exact Significance
Nominal by Nominal	Lambda	Symmetric	.054	.065	.803	.422	
		LVI_final Dependent	.000	.000	.[c]	.[c]	
		MERGED_LOD_D1 Dependent	.093	.110	.803	.422	
	Goodman and Kruskal tau	LVI_final Dependent	.029	.031		.058[d]	.079
		MERGED_LOD_D1 Dependent	.029	.031		.058[d]	.079

a. Not assuming the null hypothesis.

b. Using the asymptotic standard error assuming the null hypothesis.

c. Cannot be computed because the asymptotic standard error equals zero.

d. Based on chi-square approximation

LVI_final * E_O_LOD

Crosstab

			E_O_LOD		
			Negative	Positive	Total
LVI_final	0	Count	44	40	84
		% within LVI_final	52.4%	47.6%	100.0%
	1	Count	17	22	39
		% within LVI_final	43.6%	56.4%	100.0%
Total		Count	61	62	123
		% within LVI_final	49.6%	50.4%	100.0%

Directional Measures

			Value	Asymptotic Standard Error[a]	Approximate T[b]	Approximate Significance	Exact Significance
Nominal by Nominal	Lambda	Symmetric	.040	.090	.437	.662	
		LVI_final Dependent	.000	.000	.[c]	.[c]	
		E_O_LOD Dependent	.066	.145	.437	.662	
	Goodman and Kruskal tau	LVI_final Dependent	.007	.015		.366[d]	.439
		E_O_LOD Dependent	.007	.015		.366[d]	.439

a. Not assuming the null hypothesis.

b. Using the asymptotic standard error assuming the null hypothesis.

c. Cannot be computed because the asymptotic standard error equals zero.

d. Based on chi-square approximation

LVI_final * BOTH_LOD

Crosstab

			BOTH_LOD Negative	BOTH_LOD Positive	Total
LVI_final	0	Count	64	20	84
		% within LVI_final	76.2%	23.8%	100.0%
	1	Count	25	14	39
		% within LVI_final	64.1%	35.9%	100.0%
Total		Count	89	34	123
		% within LVI_final	72.4%	27.6%	100.0%

Directional Measures

			Value	Asymptotic Standard Error[a]	Approximate T	Approximate Significance	Exact Significance
Nominal by Nominal	Lambda	Symmetric	.000	.000	.[b]	.[b]	
		LVI_final Dependent	.000	.000	.[b]	.[b]	
		BOTH_LOD Dependent	.000	.000	.[b]	.[b]	
	Goodman and Kruskal tau	LVI_final Dependent	.016	.023		.165[c]	.195
		BOTH_LOD Dependent	.016	.023		.165[c]	.195

a. Not assuming the null hypothesis.

b. Cannot be computed because the asymptotic standard error equals zero.

c. Based on chi-square approximation

Lesion_site_cancer_only * SDC_NPY_LOD_D1

Crosstab

			SDC_NPY_LOD_D1 Negative	SDC_NPY_LOD_D1 Positive	Total
Lesion_site_canceronly	1	Count	49	24	73
		% within Lesion_site_canceronly	67.1%	32.9%	100.0%
	2	Count	29	20	49
		% within Lesion_site_canceronly	59.2%	40.8%	100.0%
	3	Count	0	1	1
		% within Lesion_site_canceronly	0.0%	100.0%	100.0%
Total		Count	78	45	123
		% within Lesion_site_canceronly	63.4%	36.6%	100.0%

Directional Measures

			Value	Asymptotic Standard Error[a]	Approximate T[b]	Approximate Significance	Exact Significance
Nominal by Nominal	Lambda	Symmetric	.011	.010	1.004	.315	
		Lesion_site_canceronly Dependent	.000	.000	.[c]	.[c]	
		SDC_NPY_LOD_D1 Dependent	.022	.022	1.004	.315	
	Goodman and Kruskal tau	Lesion_site_canceronly Dependent	.007	.015		.425[d]	.377
		SDC_NPY_LOD_D1 Dependent	.021	.015		.283[d]	.276

a. Not assuming the null hypothesis.

b. Using the asymptotic standard error assuming the null hypothesis.

c. Cannot be computed because the asymptotic standard error equals zero.

d. Based on chi-square approximation

Lesion_site_cancer_only * IKZF_SEPT9_LOD_D1

Crosstab

			IKZF_SEPT9_LOD_D1 Negative	IKZF_SEPT9_LOD_D1 Positive	Total
Lesion_site_canceronly	1	Count	45	28	73
		% within Lesion_site_canceronly	61.6%	38.4%	100.0%
	2	Count	27	22	49
		% within Lesion_site_canceronly	55.1%	44.9%	100.0%
	3	Count	0	1	1
		% within Lesion_site_canceronly	0.0%	100.0%	100.0%
Total		Count	72	51	123
		% within Lesion_site_canceronly	58.5%	41.5%	100.0%

Directional Measures

			Value	Asymptotic Standard Error[a]	Approximate T[b]	Approximate Significance	Exact Significance
Nominal by Nominal	Lambda	Symmetric	.010	.010	1.004	.315	
		Lesion_site_canceronly Dependent	.000	.000	.[c]	.[c]	
		IKZF_SEPT9_LOD_D1 Dependent	.020	.019	1.004	.315	
	Goodman and Kruskal tau	Lesion_site_canceronly Dependent	.005	.012		.568[d]	.504
		IKZF_SEPT9_LOD_D1 Dependent	.016	.012		.382[d]	.391

a. Not assuming the null hypothesis.

b. Using the asymptotic standard error assuming the null hypothesis.

c. Cannot be computed because the asymptotic standard error equals zero.

d. Based on chi-square approximation

Lesion_site_cancer_only * MERGED_LOD_D1

Crosstab

			MERGED_LOD_D1 Negative	MERGED_LOD_D1 Positive	Total
Lesion_site_canceronly	1	Count	45	28	73
		% within Lesion_site_canceronly	61.6%	38.4%	100.0%
	2	Count	24	25	49
		% within Lesion_site_canceronly	49.0%	51.0%	100.0%
	3	Count	0	1	1
		% within Lesion_site_canceronly	0.0%	100.0%	100.0%
Total		Count	69	54	123
		% within Lesion_site_canceronly	56.1%	43.9%	100.0%

Directional Measures

			Value	Asymptotic Standard Error[a]	Approximate T[b]	Approximate Significance	Exact Significance
Nominal by Nominal	Lambda	Symmetric	.019	.067	.283	.777	
		Lesion_site_canceronly Dependent	.000	.000	.[c]	.[c]	
		MERGED_LOD_D1 Dependent	.037	.128	.283	.777	
	Goodman and Kruskal tau	Lesion_site_canceronly Dependent	.016	.022		.145[d]	.162
		MERGED_LOD_D1 Dependent	.026	.022		.205[d]	.162

a. Not assuming the null hypothesis.

b. Using the asymptotic standard error assuming the null hypothesis.

c. Cannot be computed because the asymptotic standard error equals zero.

d. Based on chi-square approximation

Lesion_site_cancer_only * E_O_LOD

Crosstab

			E_O_LOD		Total
			Negative	Positive	
Lesion_site_canceronly	1	Count	39	34	73
		% within Lesion_site_canceronly	53.4%	46.6%	100.0%
	2	Count	22	27	49
		% within Lesion_site_canceronly	44.9%	55.1%	100.0%
	3	Count	0	1	1
		% within Lesion_site_canceronly	0.0%	100.0%	100.0%
Total		Count	61	62	123
		% within Lesion_site_canceronly	49.6%	50.4%	100.0%

Directional Measures

			Value	Asymptotic Standard Error[a]	Approximate T[b]	Approximate Significance	Exact Significance
Nominal by Nominal	Lambda	Symmetric	.045	.075	.586	.558	
		Lesion_site_canceronly Dependent	.000	.000	.[c]	.[c]	
		E_O_LOD Dependent	.082	.134	.586	.558	
	Goodman and Kruskal tau	Lesion_site_canceronly Dependent	.007	.015		.414[d]	.360
		E_O_LOD Dependent	.015	.015		.401[d]	.409

a. Not assuming the null hypothesis.

b. Using the asymptotic standard error assuming the null hypothesis.

c. Cannot be computed because the asymptotic standard error equals zero.

d. Based on chi-square approximation

Lesion_site_cancer_only * BOTH_LOD

Crosstab

			BOTH_LOD		Total
			Negative	Positive	
Lesion_site_canceronly	1	Count	55	18	73
		% within Lesion_site_canceronly	75.3%	24.7%	100.0%
	2	Count	34	15	49
		% within Lesion_site_canceronly	69.4%	30.6%	100.0%
	3	Count	0	1	1
		% within Lesion_site_canceronly	0.0%	100.0%	100.0%
Total		Count	89	34	123
		% within Lesion_site_canceronly	72.4%	27.6%	100.0%

Directional Measures

			Value	Asymptotic Standard Error[a]	Approximate T[b]	Approximate Significance	Exact Significance
Nominal by Nominal	Lambda	Symmetric	.012	.012	1.004	.315	
		Lesion_site_canceronly Dependent	.000	.000	.[c]	.[c]	
		BOTH_LOD Dependent	.029	.029	1.004	.315	
	Goodman and Kruskal tau	Lesion_site_canceronly Dependent	.005	.012		.544[d]	.445
		BOTH_LOD Dependent	.026	.012		.209[d]	.220

a. Not assuming the null hypothesis.

b. Using the asymptotic standard error assuming the null hypothesis.

c. Cannot be computed because the asymptotic standard error equals zero.

d. Based on chi-square approximation
